# The frequency of reduction loss after arthroscopic fixation of acute acromioclavicular dislocations using a double-button device, and its effect on clinical and radiological results

**DOI:** 10.1186/s13018-020-01674-x

**Published:** 2020-04-08

**Authors:** Engin Çarkçı, Ayşe Esin Polat, Tahsin Gürpınar

**Affiliations:** 1 Department of Orthopedics and Traumatology, Istanbul Training and Research Hospital, 34098 Fatih, Istanbul, Turkey; 2 Department of Orthopaedics and Traumatology, Dr. Akçiçek State Hospital, 99300 Kyrenia, Cyprus

**Keywords:** Acromioclavicular joint, Dislocation, Endobutton, Arthroscopy, Coracoclavicular distance

## Abstract

**Background:**

The aim of this study was to investigate the effect of reduction loss of more than 3 mm on clinical and radiological results after at least 2 years of follow-up after arthroscopic fixation of acute acromioclavicular joint dislocations using a double-button device.

**Methods:**

Thirty-six patients who had acute (< 3 weeks old), type III or V acromioclavicular (AC) joint dislocations underwent arthroscopic fixation of the AC joint using a double-button device. Clinical and radiological evaluations were performed at preoperative, postoperative first day, 3 months and last follow-up. When the coracoclavicular (CC) distances of patients at the last follow-up were compared to the early postop CC distances, those with a difference of 3 mm or less were grouped as group A and those with a difference of more than 3 mm were grouped as group B.

**Results:**

There was no statistically significant difference between the groups in terms of age, gender, follow-up time, time from injury to surgery, return to work, and distribution of Rockwood classification. Pre-operative CC distance was reduced from 18.7 ± 3.5 to 8.5 ± 0.6 in the early postoperative period. Anatomic reduction was achieved in all patients compared with the unaffected side (CC distance 8.6 ± 0.7). However, the CC distance increased to 9.9 ± 1.5 at the third-month follow-up and increased to 11 ± 2.7 at the last follow-up. There were no significant Constant score differences between the groups in the preoperative and last follow-up periods (*p* > 0.05). At the last follow-up, the mean Acromioclavicular Joint Instability (ACJI) score of group A was 84.4 ± 8, whereas it was 68.3 ± 8.3 for group B, and the difference was statistically significant (*p* < 0.01). Furthermore, the subjective evaluation and aesthetic subjective satisfaction values of group B were lower than group A (*p* < 0.01).

**Conclusions:**

Reduction loss of more than 3 mm was observed in 25% of patients after arthroscopic fixation of acute acromioclavicular dislocations using a double-button device. Although this loss did not create a statistically significant difference in Constant scores, AC joint-specific tests such as ACJI, subjective evaluation, and aesthetic subjective satisfaction values were significantly impaired.

## Background

Acromioclavicular (AC) joint dislocation is not a common orthopaedic injury with the incidence of 1.8 per 10,000 per year [1]. Rockwood divided AC dislocations into six types and according to current recommendations; types IV through VI are managed by surgical reconstruction and treatment of type III should be individualized based on the patient’s demands, activity level and response to conservative treatment. There are several methods used for fixation like K-wires, hook plate, Bosworth screw, Weaver-Dunn and resection of the lateral end of the clavicle, but there is no gold standard procedure [2–4]. Recently, arthroscopic techniques have been successfully proposed to treat AC joint dislocations [5–7]. However, despite continuous development in surgical management, current techniques are still associated with many significant complications including under-correction, loss of reduction, coracoid fractures, clavicle fractures, infection and adhesive capsulitis [8].

In order to solve complications such as reduction loss, techniques including opening of a second tunnel [9], supporting the existing tunnel with anchor to the coracoid [10] and using tendon graft [11] have been proposed by different authors. However, none of these techniques have been widely accepted. Furthermore, these additional procedures make the cases more complex and harder to manage.

We have been using an adjustable loop double button device (Smith Nephew) for arthroscopic fixation of acute AC joint disruption (< 3 weeks) without additional augmentation for the last 4 years in our hospital. Clinical and radiological outcomes of patients and the loss of reduction with this technique have not been widely studied in the literature. The aim of this study was to investigate the effect of reduction loss of more than 3 mm on clinical and radiological results after at least 2 years of follow-up after arthroscopic fixation of acute acromioclavicular joint dislocations using a double-button device.

## Methods

The study protocol was approved by the institutional review board of our hospital (2019 no: 2051), and we retrospectively reviewed the medical records of patients with AC joint dislocation who were treated arthroscopically in our institution between July 2015 and July 2017. Only acute cases (< 3 weeks old), type III or V AC joint dislocations who had a minimum 2-year follow-up with availability of all records were included in the study. Patients with other dislocation types, presence of acromial, coracoid or clavicular fracture, with associated rotator cuff, labral, glenohumeral or biceps tendon injury diagnosed during the arthroscopic procedure were excluded. A total of 41 patients met the inclusion criteria, but 5 patients were lost at the last follow-up. Finally, the remaining 36 patients were enrolled in this study. The anteroposterior radiographic views of the bilateral AC joints were taken of all patients in order to diagnose and evaluate each AC joint dislocation before operating. All operations were performed by the same senior surgeon (T.G.) with the technique described below. If there was a difference of more than 3 mm between the coracoclavicular (CC) distance at last follow-up compared to early postop, this was considered as reduction loss. When the patients’ CC distances at last follow-up were compared to the early postop CC distance, those with a difference of 3 mm or less were grouped as group A and those with a difference of more than 3 mm were grouped as group B.

### Surgical technique

Patients were placed in the beach chair position under general anaesthesia. After establishment of the standard posterior portal, an anterior portal through the rotator interval using an outside-in technique was opened. The glenohumeral joint was first evaluated with a 30° scope, and the rotator interval was opened using a radiofrequency device. After that, the scope was changed to 70° and the base of the coracoid was exposed using the radiofrequency device. A 2–3-cm incision was made over the clavicle 3–4 cm far from the AC joint, and the centre of the clavicle was identified. The drill guide was inserted through the anterior portal, and the guide tip was positioned under the coracoid base. A 2.4-mm guide pin was inserted from the clavicle to the coracoid. A 4-mm cannulated drill was then passed over the pin and through the coracoid. The pin was then removed, and the drill was left in situ. A Number 3 Prolene suture was passed through the drill and taken out through the anterosuperior portal using an arthroscopic grasper, leaving the suture loop superiorly. The drill was then removed, and the Prolene was used to pass the ethibond attached to one side of the double loop endobutton system. The traction was applied from the anterior portal, and a grasper was also used to pass the endobutton through the coracoid. Once the button passed under the coracoid, the trailing suture was used to flip it, locking it under the bone. The clavicle was then reduced by the surgical assistant. The sutures were tied over the top of the superior button. The wounds were then closed in layers.

A shoulder sling was worn for 4 weeks. Passive range of motion exercise began 2 weeks after the operation. Strengthening exercises began at 8 weeks, and the patients were allowed to perform heavy weight lifting at 3 months after the operation. Contact sports were not permitted until 6 months after the operation.

### Clinical evaluation

Patients’ demographics, trauma mechanism, associated injuries and time from injury to surgery were questioned and noted preoperatively. Clinical follow-up was performed preoperatively and at last follow-up. Radiological follow-up was performed preoperatively, early postoperatively, at 3 months and at the last follow-up. Patients were clinically evaluated using preoperative and postoperative Constant, subjective evaluation and aesthetic subjective satisfaction at last follow-up. The patients were questioned to subjectively evaluate the injured shoulder as excellent, good, fair and poor. Furthermore, patients were asked to describe their aesthetic subjective satisfaction as highly satisfied, satisfied, poorly satisfied and unsatisfied. In addition, we used the Acromioclavicular Joint Instability Score (ACJI) [12] in order to evaluate the AC joint specifically, since Constant is not specific to AC joint. The ACJI evaluates 5 variables including pain, daily living activities, cosmesis, function and radiological evaluation.

Radiological evaluation included anteroposterior (AP), axillar and Zanca views of each shoulder and bilateral stress radiographs. Measurements were performed on standard AP shoulder X-rays which are centred 2 cm inferior to the lateral clavicle at the level of glenohumeral joint. Alexander view radiographs were obtained in order to calculate the ACJI score at the last follow-up. Dislocations were graded according to the Rockwood classification. CC distance was calculated at preoperative, early postoperative, postoperative 3 months and postoperative last follow-up on AP X-rays as the perpendicular distance between the uppermost point of the superior cortex of the coracoid and the undersurface of the clavicle and then compared with the normal side. Mean values were recorded in millimetres for the vertical distance using a digital caliper in picture archiving and communication system by two different orthopaedic surgeons and averaged. ∆ is the side to side difference in percentage of a distance measure at last follow-up. The imaging studies were also examined for any loss of reduction in the AC joint, or other possible complications, such as fracture, hardware migration or heterotopic ossification. Demographic characteristics and functional and radiological scores of patients between groups were compared.

### Statistical analysis

Mean, standard deviation, median lowest, highest, frequency and ratio values were used in descriptive statistics of the data. The chi-square test was used for the analysis of qualitative independent data, and the Fisher test was used when the chi-square test conditions were not met. The Mann-Whitney test was used to analyze quantitative independent data. SPSS 18.0 program was used in the analysis.

## Results

The mean age was 30.6 ± 7.51 years (range, 20–52). Thirty-two patients were male and 4 were female. The mean follow-up was 31.4 ± 5.9 months (range, 24–48). According to the Rockwood classification, there were 14 type III and 22 type V dislocations. Mean time from injury to surgery was 6.8 ± 3.8 days (range, 2–14). The mean time to return to work was 11.2 ± 2.9 weeks (range, 8–20). When trauma mechanisms are examined, 15 patients were injured while doing sports, 14 patients were injured after a traffic accident, 5 patients were injured after falling from height and 2 patients were injured during a fight. When the accompanying traumas were examined, 4 patients had thorax trauma, 2 patients had head trauma and 1 patient had lumbar fractures (Table 1).

**Table 1:** Demographic characteristics of patients

		Min–max	Median	Mean ± sd/*n* (%)	
Age		20–52	29.00	30.56 ± 7.51	
Sex	Female			4	11.1%
	Male			32	88.9%
Follow-up time (months)		24–48	30.00	31.42 ± 5.89	
Trauma mechanism	Traffic accident			14	38.9%
	Sports			15	41.7%
	Fall from height			5	13.9%
	Fight			2	5.5%
Associated Injuries	Lumber fracture			1	14.3%
	Head trauma			2	28.6%
	Thorax trauma			4	57.1%
Time from injury to surgery (days)		2–14	5.00	6.78 ± 3.85	
Rockwood classification	Type III			14	38.9%
	Type V			22	61.1%
Return to work (months)		8–20	10.00	11.17 ± 2.88	

There was an improvement in the mean Constant–Murley Shoulder Score from 27.8 ± 7.9 (range, 18–46) pre-operatively to 92 ± 4.6 (range 82–100) post-operatively, which was statistically significant (*p* < 0.05). The mean ACJI score was 80.4 ± 10.6 at the last follow-up. According to the subjective evaluation, 72.2% of the patients rated the result as excellent and 27.8% of the patients rated it as good. When aesthetic subjective satisfaction was questioned, 72.2% of the patients stated that they were highly satisfied, 19.5% were satisfied and 8.3% expressed low satisfaction. When the patients were examined radiologically, the pre-operative CC distance was reduced from 18.7 ± 3.5 to 8.5 ± 0.6 in the early postoperative period. Anatomic reduction was achieved in all patients compared with the unaffected side (CC distance 8.6 ± 0.7). However, the CC distance increased to 9.9 ± 1.5 at the third-month follow-up and increased to 11 ± 2.7 at the last follow-up. The mean loss of reduction was 27.4% ± 28.6% (range, 0–100) compared to the unaffected side at the last follow-up (Table 2).

**Table 2:** Clinical and radiological results of patients

		Min–max	Median	Mean ± sd/*n* (%)	
Constant score					
Preop		18–46	26.00	27.78 ± 7.93	
Last follow-up		82–100	92.00	92.03 ± 4.62	
Last follow-up ACJI score		60–95	83.50	80.39 ± 10.62	
Subjective evaluation	Good			10	27.8%
	Excellent			26	72.2%
Aesthetic subjective satisfaction	Poorly satisfied			3	8.3%
	Satisfied			7	19.5%
	Highly satisfied			26	72.2%
CC distance (mm)					
Unaffected side		8–10	9.00	8.64 ± 0.68	
Preop		13–24	19.00	18.72 ± 3.53	
Early post-op		8–10	8.00	8.47 ± 0.61	
3 months		8–14	9.00	9.89 ± 1.47	
Last follow-up		8–17	10.00	11.03 ± 2.71	
Compared unaffected side at last follow-up (%)		0–100	21.10	27.43 ± 28.55	

There was no statistically significant difference between the groups in terms of age, gender, follow-up time, time from injury to surgery, return to work and distribution of Rockwood classification. There were no significant Constant score differences between the groups in the preoperative and last follow-up periods (*p* > 0.05). At the last follow-up, the mean ACJI score of group A was 84.4 ± 8, whereas it was 68.3 ± 8.3 for group B, and the difference was statistically significant (*p* < 0.01). Furthermore, there was a significant difference between groups in terms of subjective evaluation and aesthetic subjective satisfaction (*p* < 0.01). There was no statistically significant difference between the CC distance of groups in the preoperative and early postoperative periods. On the other hand, it was found that CC distance was statistically lower in group A than in group B at 3 months and last follow-up control. When CC distances were compared to unaffected side at the last follow-up, 13.2% ± 10.6 (range, 0–33.3) of reduction loss was observed in group A and 70% ± 2.2 (range, 33.3–100) of reduction loss was observed in group B. This difference is statistically significant (*p* < 0.01) (Table 3). No complications including tunnel malposition or early implant failure leading to revision were observed. In addition, no fractures of the coracoid or the clavicle were encountered.

**Table 3:** Comparison of demographic, clinical and radiological outcomes between groups

		Group A (*n* = 27)		Group B (*n* = 9)		*p*
		Mean ± sd/*n* (%)	Median	Mean ± sd/*n* (%)	Median	
Age		29.89 ± 8.13	27.00	32.56 ± 5.13	33.00	0.133^m^
Sex	Female	3	11.1%	1	11.1%	0.999^f^
	Male	24	88.9%	8	88.9%	
Follow-up time		32.11 ± 6.49	30.00	29.33 ± 2.92	30.00	0.442^m^
Time from injury to surgery		6.70 ± 3.89	5.00	7.00 ± 3.94	7.00	0.825^m^
Return to work		10.59 ± 2.41	10.00	12.89 ± 3.62	12.00	0.068^m^
Rockwood classification	Type III	12	44.4%	2	22.2%	0.432^f^
	Type V	15	55.6%	7	77.8%	
Constant						
Preop		28.48 ± 7.99	27.00	25.67 ± 7.81	22.00	0.226^m^
Last follow-up		92.48 ± 4.78	92.00	90.67 ± 4.03	92.00	0.329^m^
Last follow-up ACJI score		84.41 ± 7.99	86.00	68.33 ± 8.28	64.00	**0.000**^m^
CC distance						
Unaffected side		8.52 ± 0.64	8.00	9.00 ± 0.71	9.00	0.067^m^
Preop		18.48 ± 3.69	19.00	19.44 ± 3.09	20.00	0.508^m^
Early post-op		8.44 ± 0.64	8.00	8.56 ± 0.53	9.00	0.462^m^
3 months		9.22 ± 0.80	9.00	11.89 ± 1.17	12.00	**0.000**^m^
Last follow-up		9.63 ± 1.01	10.00	15.22 ± 1.56	15.00	**0.000**^m^
Compare unaffected side at last follow-up (%)		13.24 ± 10.60	12.50	70.02 ± 21.94	75.00	**0.000**^m^
Subjective evaluation	Good	4	14.8%	6	66.7%	**0.006**^f^
	Excellent	23	85.2%	3	33.3%	
Aesthetic subjective satisfaction	Poorly satisfied	0	0.0%	3	33.3%	**0.012**^f^
	Satisfied + highly satisfied	27	100.0%	6	66.7%	

^m^Mann-Whitney U Test

^f^Fisher’s Exact Test

## Discussion

There is no consensus regarding the ideal surgical technique for AC joint dislocation [4]. Different open and arthroscopic techniques have been proposed in the literature. Recently, clinical and biomechanical studies have shown that suture button fixations are effective for coracoclavicular reconstruction of the joint [5, 12, 13]. In this procedure, the CC ligaments are expected to heal since the AC joint is kept reduced and the AC and CC ligament remnants are brought into contact. In a systemic meta-analysis, loop fixation devices were found to have higher shoulder function scores and lower postoperative pain [14]. Another study comparing the results of loop button fixation and Bosworth screw fixation showed that more treatment satisfaction is achieved with loop fixation [15]. Our study showed that arthroscopic fixation of acute acromioclavicular joint disruptions (type III and V) by using a double-button device achieves satisfactory outcomes. Our findings are consistent with other authors reporting satisfactory outcomes using similar techniques and device.

Biomechanical studies have shown that button systems provide excellent supero-inferior stability with load to failure higher than native ligament [16, 17]. However, horizontal instability in AC dislocations is increasingly being treated with AC repair, with an additional acromioclavicular sling [18]. AC repair was not performed in any of the patients in this study group since it requires wider dissection and longer surgery, it has higher morbidity and the additional stability is questionable. In a cadaver study, Weiser et al. did not observe any additional stability with direct AC repair [19].

The patients in our study were operated by using a single tunnel to avoid the risk of coracoid fracture. In a biomechanical study by Beitzel et al. [13], there was no difference in stability between the single and double device; nevertheless, the coracoid fracture incidence was much higher with the double device. Likewise, a recent study demonstrated that single tunnel reconstruction demonstrates similar biomechanical properties to the intact state and double tunnel reconstruction [9].

Coracoid fractures have been reported as a potential complication in the literature [20]. We did not have any such complications. We believe that appropriate visualization of the inferior surface of coracoid process and drilling with a 4-mm drill close to the base of the coracoid is essential. In a cadaveric study, Rylander et al. [21] showed that a 4-mm tunnel technique is significantly stronger than a 6-mm tunnel technique when using a transcoracoid reconstruction technique. In addition, tunnel placement is also a significant factor to avoid coracoid fractures and failure of fixation. In a recent study, higher peak load to failure was found when a centre-centre or medial-centre tunnel orientation was performed during drilling [22].

Recently, some authors have suggested CC ligament reconstruction by using tendon grafting [23]. However, in another study, 47% reduction loss and 20% complications were seen after CC ligament reconstruction with an autologous tendon graft, which adversely affected the results [11]. Many previous open procedures treating the AC joint dislocation without tendon reconstruction, such as hook plate fixation, K-wire and Bosworth type screw fixation, have shown good results after the implants were removed. Therefore, we believe it is viable to do the CC ligament reconstruction without ligamentous augmentation.

The most commonly reported complication after AC joint reconstruction is loss of reduction. In this study, 9 of 36 patients had a reduction loss greater than 3 mm CC distance compared with the unaffected side. There are some possibilities for the reduction loss. In patients operated with the single flip button device technique, reduction loss has been shown to be caused by a longer duration between injury and treatment of more than 5 days and poor quality of initial reduction (more than 2 mm CC distance difference in early postop radiological examination) [24]. In our study, no statistically significant difference was observed between groups with and without reduction loss in terms of time from injury to treatment. In addition, early radiological examination revealed a CC difference of less than 2 mm in all patients. Our adjustable loop system only mimics one component of the CC ligament, and the AC ligament was not also reconstructed. Undue forces on the AC joint may cause instability and damage the healing process of the CC ligaments. In addition, excessive force on the bone metal button interface may result in bone erosion, clavicular tunnel widening and cause loss of reduction [25]. Another reason could be loosening of the adjustable loop system. Zhang et al. [26] showed 25% fixation failure rate within 3–6 months after CC fixation using a similar suspensory fixation device. In different series, reduction loss varies; however, good or excellent outcomes are reported regardless of fixation loss. Murena et al. [27] reported outcomes of 16 patients with AC dislocation treated by the double flip button technique. Although 25% of the patients had a reduction loss, the average Constant score was 97.

De Carli et al. compared the functional results of patients with double-button fixation and conservative treatment of 3rd degree AC joint dislocations. According to this study, although there was no difference in terms of non-AC joint-specific objective scores in the surgical group, a statistically significant difference was found in the AC joint-specific objective measurements, subjective evaluation of patients and aesthetic satisfaction [28]. In our study, although there was no difference in non-AC joint-specific objective scores in the group without loss of reduction, a statistically significant difference was found in AC joint-specific objective scores, subjective assessment and aesthetic satisfaction. Therefore, the authors believe that reduction maintaining is crucial for excellent functional and aesthetic results after fixation of the AC joint with a double-button device.

There are some limitations to this study. First, the number of patients was small. Second, our follow-up time was not sufficient to make a conclusion about the incidence of post-traumatic arthritis; however, we were able to show early results and complications since most of the reduction loss occurs in a short time.

## Conclusion

Reduction loss of more than 3 mm was observed in 25% of patients after arthroscopic fixation of acute acromioclavicular dislocations using a double-button device. Although this loss did not create a statistically significant difference in Constant scores, AC joint-specific tests such as ACJI, subjective evaluation and aesthetic subjective satisfaction values were significantly impaired.

## Data Availability

The data of this study were real and were performed in SPSS (version 22.0). The statistical results of the data are presented in the main paper. All of the data are available in contact with the correspondence author.

## Abbreviation

AC: Acromioclavicular
CC: Coracoclavicular
ACJI: Acromioclavicular joint instability
